# A Feasibility Study on HPMC-Improved Sulphoaluminate Cement for 3D Printing

**DOI:** 10.3390/ma11122415

**Published:** 2018-11-29

**Authors:** Zhu Ding, Xiaodong Wang, Jay Sanjayan, Patrick X.W. Zou, Zhi-Kun Ding

**Affiliations:** 1 Guangdong Provincial Key Laboratory of Durability for Marine Civil Engineering, College of Civil Engineering, Shenzhen University, Shenzhen 518060, China; zding@szu.edu.cn (Z.D.); ddzk@szu.edu.cn (Z.-K.D.); 2 Center for Sustainable Infrastructure, School of Engineering, Faculty of Science, Engineering and Technology, Swinburne University of Technology, Melbourne 3122, Victoria, Australia; jsanjayan@swin.edu.au (J.S.); pwzou@swin.edu.au (P.X.W.Z.)

**Keywords:** 3D construction printing, sulphoaluminate cement, hydroxypropyl methylcellulose (HPMC), shape retainability, hydration, compressive strength

## Abstract

A novel 3D printing material based on hydroxypropyl methylcellulose (HPMC)—improved sulphoaluminate cement (SAC) for rapid 3D construction printing application is reported. The hydration heat, setting time, fluidity of paste and mortar, shape retainability, and compressive strength of extruded SAC mortar were investigated. HPMC dosage, water-to-cement (W/C) ratio, and sand-to-cement (S/C) ratio were studied as the experimental parameters. Hydration heat results reveal HPMC could delay the hydration of SAC. The initial and final setting time measured using Vicat needle would be shortened in the case of W/C ratio of 0.3 and 0.35 with HPMC dosage from 0.5% to 1.5%, W/C ratio of 0.40 with HPMC dosage of 0.5%, 0.75%, and 1.5%, and W/C ratio of 0.45 with HPMC dosage of 0.45, or be extended in the case of W/C ratio of 0.4 with HPMC dosage of 1.0% and W/C ratio of 0.45 with HPMC dosage from 0.75% to 1.5%. Fluidity measurement shows HPMC significantly improves the shape retainability. Furthermore, the addition of HPMC remarkably increased the compressive strength of extruded mortar. The results showed that HPMC could be used to prepare 3D printing SAC having satisfactory shape retainability, setting time and compressive strength.

## 1. Introduction

In recent years, 3D printing technology has been used for buildings and other structures, and related studies have been conducted to develop 3D concrete printing which is based on layer-by-layer printing process [1,2,3,4,5,6,7,8,9,10,11]. Developing a suitable 3D printing building material is one of the most pressing problems for 3D concrete printing [2,5,12]. Some of the most critical issues for 3D printing of building materials are the shape retainability (also known as shape stability [13] and green strength [14]), workability, and early strength development.

When the material is printed and deposited, it should maintain its extruded shape without flowing away. The application of an additive is an effective method to enhance the shape stability of printed cement-based materials. Some researchers have aimed at engineering the rheology of printed materials by modifying the mix design [15,16,17] and changing the mixing method [17,18,19]. Decreasing the water-to-cement ratio induces an increment in rheological parameters (yield stress and viscosity), making the mix sticky [16,17,20]. Increasing an aggregate content could bring the improvement in concrete rheological parameters [21]. The addition of chemical admixtures is also an effective method to modify the rheological properties. The application of superplasticizer could reduce the yield stress [17]. The addition of alginate could help the earth-based materials to develop sufficient green strength in a short period of time [14].

Hydroxypropyl methylcellulose (HPMC) is widely used as a thickener to modify rheological properties by saving cement-based materials from bleeding and segregation [22,23,24,25,26,27]. Some experimental researches [27,28,29,30] have proved that HPMC has excellent water retention property in cement-based materials. Zhi et al. [23] found that HPMC could enhance the viscosity of gypsum slurry. Deng et al. [24] reported that plasticizer HPMC was one of the most important factors for designing 3D printed alumina slurry. Liu [31] stated that silanized HPMC significantly improved the injectability of calcium phosphate cement paste by increasing the viscosity due to the gelation of HPMC, producing a 3D network. Paiva et al. [32] observed that the yield stress of mortar showed an initial decrease followed by an increase when the dosage of HPMC changed from 0.05% to 0.20%. Poinot et al. [29] reported that HPMC can decreased both the fluidity index and yield stress of Portland cement mortars.

Sulphoaluminate cement (SAC) exhibits fast setting and high early strength to support efficient and rapid construction; thus, SAC is advantageous to be utilized in 3D printing applications [33,34,35,36]. In comparison to ordinary Portland cement, SAC also offers several advantages, such as relatively low energy consumption and carbon dioxide emission, excellent durability, and resistance to freezing-thawing [37,38,39,40,41]. Moreover, SAC is suitable to be used in cold environment constructions [33].

In this study, a 3D printing material was developed based on SAC by incorporating HPMC. The setting time, fluidity and hydration heat of fresh SAC paste was tested by changing the water-to-cement (W/C) ratio and HPMC dosage. The fluidity of fresh SAC mortar was measured by optimizing HPMC dosage, W/C ratio, and sand-to-cement (S/C) ratio by mass. Furthermore, the early strength of the SAC mortar was tested for a series of HPMC dosages.

## 2. Materials and Methods

### 2.1. Materials

The sulphoaluminate cement (SAC) with a specific surface area of 367 m^2^∙kg^−1^ was obtained from a commercial cement producer. The chemical composition of SAC analyzed by X-ray fluorescence (XRF) is provided in Table 1. The HPMC with a viscosity of 200,000 mPa⋅s was used as a modifying agent to improve shape stability of SAC-based materials. The results of methoxy and hydroxypropyl assays for HPMC were 25.8% and 10.5%, respectively. ISO standard sand, produced by Xiamen Isosand Co., Ltd. (Xiamen, China), was used as a fine aggregate in this study.

### 2.2. Preparation of SAC Paste Samples

SAC pastes were prepared by mixing SAC, HPMC, and water at various W/C ratios and HPMC dosages (wt.% cement). The designed paste proportions that are shown in Table 2 were investigated to assess the effect of W/C ratio and HPMC content on the setting time and fluidity.

### 2.3. Preparation of SAC Mortar Samples

The SAC mortar was prepared with SAC, HPMC, sand, and water at various HPMC dosages (wt.% cement), sand-to-cement (S/C) ratio, and W/C ratio, using the mixing proportions shown in Table 3.

### 2.4. Test Methods

#### 2.4.1. X-Ray Diffraction Analysis

X-ray diffraction (XRD) was used to analyze the SAC clinker and hydrated SAC. Samples were grounded manually in an agate mortar to pass through a 400-mesh sieve. XRD patterns were obtained using a Bruker D8 Advance Diffractometer with Cu Kα-radiation at 2θ angles varying from 5° to 80° with an interval of 0.02°.

#### 2.4.2. Hydration Heat

The hydration heat was measured using an isothermal heat flow calorimeter controlled by a computer (ToniCAL Trio 7338, Toni Technik, Berlin, Germany) at 25 °C. The heat release rate and the accumulative heat release of hydration during the early stage was evaluated. About 10 g of cement was weighed using electronic analytical balance with accuracy of 0.0001 g, and then was placed in a glass test tube followed by being put inside the calorimeter. After the sample equilibration finished, water was injected into cement inside the calorimeter. At the same time, the heat release started being recorded.

#### 2.4.3. Setting Time and Fluidity

The setting time and fluidity of fresh SAC paste and mortar were tested at room temperature (25 °C). The setting time of fresh SAC paste was determined using Vicat apparatus according to GB/T 1346–2011 (ISO 9597:2008). When the paste was close to the initial or final setting, the testing was carried out every one minute

The fluidity of fresh SAC paste was measured according to GB/T 8077–2000. The fluidity of SAC mortar was determined using a flow table according to GB/T 2419-2005. The inner diameter of the upper and lower mouth of the mold used to measure the mortar fluidity was 70 mm and 100 mm, respectively. The frequency of the jolting apparatus was once per second, and 25 jolting were completed in 25 ± 1 s. In addition to fluidity value, the height of the mortar was recorded after removing the mold before jolting (initial height), and after jolting (final height).

#### 2.4.4. Compressive Strength

The fresh mortar was extruded into cubic molds of 30 mm × 30 mm × 30 mm in size. Consequently, the molds with fresh SAC mortar were sealed with a plastic film to prevent water evaporation and then put in a standard maintenance box of cement with a temperature of 20 ± 1 °C and relative humidity of ≥90%. The SAC mortar specimens were removed carefully after 50 min and cured in the maintenance box until the intended test age was reached. Mortar specimens were cured for 1 h and 2 h. The compressive strength of SAC mortar specimens was determined using a computer-controlled compression testing machine with a loading rate in the range of 2.0–2.4 kN/s. The average compressive strength of three replicates was reported with an accuracy of 0.1 MPa.

#### 2.4.5. Microstructure

The hardened paste and mortar with and without HPMC were characterized by using scanning electron microscope (SEM) (Quanta TM 250 FEG, FEI, Hillsboro, OR, USA) to analyze the microstructure transformation resulted from the addition of HPMC. The hardened paste and mortar were cut into pieces which were dried in a vacuum drying oven at 30 °C for 12 h. Before the SEM test, the dried pieces were cut apart into small pieces with size of about 5 mm × 5 mm × 5 mm. For each paste and mortar, one piece was selected to be coated with gold film by gold spraying with placing the fresh section surface up. Then, the fresh section surface was observed by using SEM.

## 3. Results and Discussion

### 3.1. XRD Analysis of SAC and the Hydrated Samples

**Figure 1:**
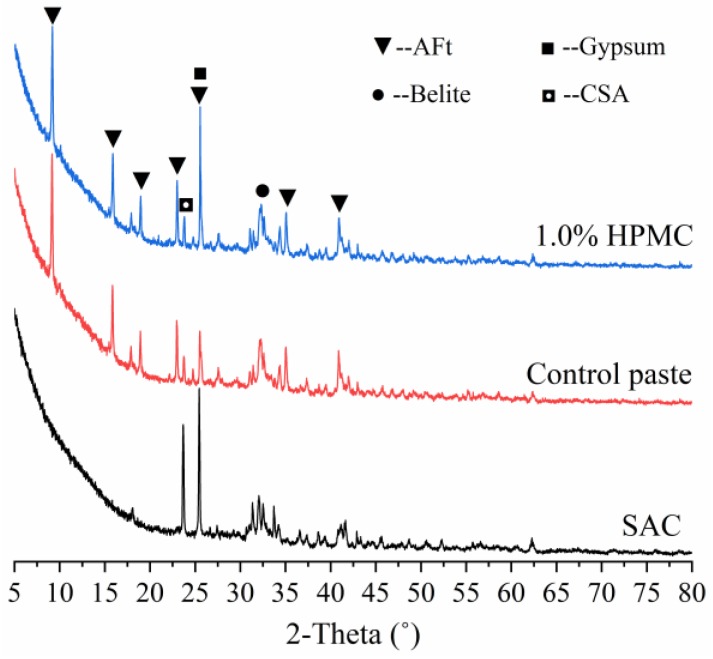
XRD patterns of SAC, control SAC paste and 1.0% HPMC SAC paste with W/C ratio of 0.40 at hydration time of 1 h.

Figure 1 shows the XRD patterns of SAC raw material and SAC pastes with and without HPMC at the hydration time of 1 h with W/C ratio of 0.40. According to Figure 1, at the hydration time of 1 h, most of the calcium sulphoaluminate (CSA), the main phase in SAC, has been hydrated to form a large amount of hydration products (ettringite, AFt) to produce high early strength. The high early strength of SAC is an invaluable advantage for its deployment as 3D printing material to realize rapid manufacturing.

### 3.2. Influence of HPMC on Hydration Heat of SAC

**Figure 2:**
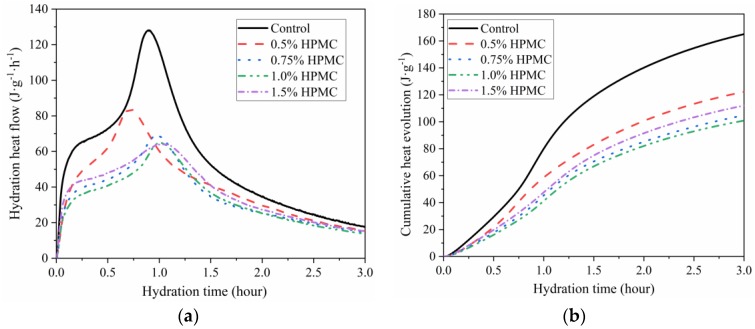
Hydration heat calorimetry of fresh mixed SAC paste with and without HPMC at W/C ratio of 0.40: (**a**) Hydration heat flow during first 3 h of hydration; (**b**) cumulative hydration heat during first 3 h of hydration.

Figure 2 shows the hydration heat of fresh SAC paste with and without HPMC at W/C ratio of 0.40. The addition of HPMC resulted in the decrease in hydration heat flow. The heat flow peak was diminished and impeded by the increase of HPMC dosage from 0.5% to 1.5%. An initial decline followed by an increase in cumulative heat evolution was observed with increasing HPMC dosage. The cumulative heat release decreased with increasing HPMC dosage from 0 to 1.0%, while the total heat of SAC pastes with 1.5% HPMC increased to be greater than that of the SAC paste with 0.75% and 1.0% HPMC. These results indicate that the addition of HPMC could delay the hydration of cement due to the absorption of water. The delay effect became stronger with the increase in HPMC dosage from 0.5% to 1.0%. However, when the content of HPMC increased to 1.5%, the delay effect was weaker than for 0.75% HPMC.

### 3.3. Influence of W/C Ratio and HPMC Content on the Setting Time of SAC

The 3D printing of cement-based materials requires a reasonable setting time that is neither too short nor too long. Enough time should be provided to mixing, transporting, and printing materials in proper order to avoid extrudability loss of printing materials even becoming hardened in the tube or printing nozzle before printing. In general, the cementitious materials begin to lose their extrudability earlier than the initial setting time [13]. Hence, the initial setting time should be tested and tuned to avoid this problem. Meanwhile, after being printed and deposited, the materials should become hardened to provide enough strength to support the following printed materials. This process should be relatively fast to further boost the printing efficiency [42] which is one of the greatest advantages of 3D construction printing. In other words, the time gap between the initial and final setting time should be satisfactory.

**Figure 3:**
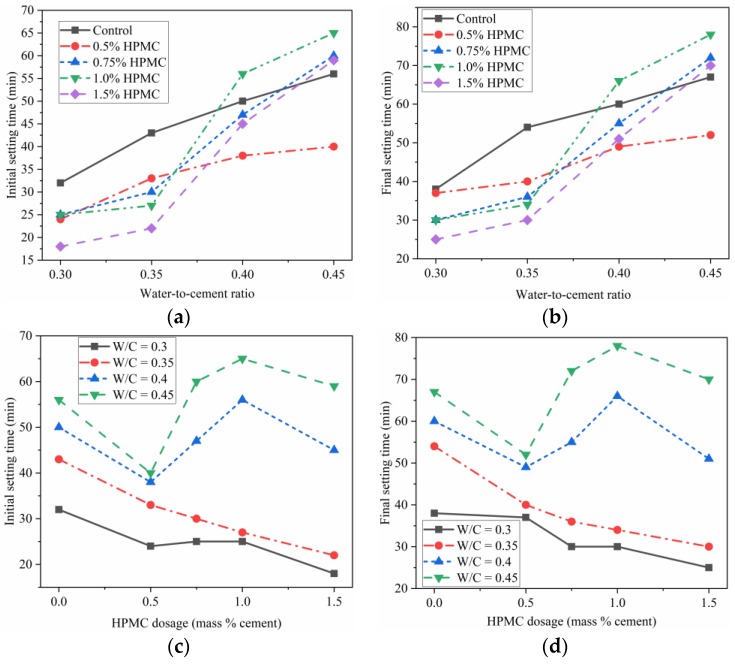
Setting time of the fresh mixed SAC paste with and without HPMC at different W/C: (**a**) initial setting time are plotted against W/C; (**b**) final setting time plotted against W/C; (**c**) initial setting time plotted against HPMC dosage; (**d**) final setting time plotted against HPMC dosage.

The setting time of SAC paste with and without HPMC at various W/C ratios were assessed. The influence of W/C ratio and addition of HPMC on the setting time of paste is shown in Figure 3. Table 4 lists the extended and shortened time of the initial and final setting times of the paste with HPMC compared to the control sample. The initial setting time of SAC with W/C ratio from 0.30 to 0.45 and HPMC content from 0 to 1.5% ranged from 18 min to 65 min, and the final setting time ranged from 25 min to 78 min. As is evident from Figure 3, the initial and final setting times were extended with the increase of W/C ratio, while HPMC had a more intricate effect on the setting time of the paste with different W/C ratio. For both the control paste and paste with 0.5% HPMC, a higher W/C ratio bring a lower increase rate of the setting time (Figure 3a,b). On the other hand, for the pastes blended with 0.75%, 1.0% and 1.5% HPMC, the change in W/C ratio from 0.35 to 0.40 induced the higher increase rates of both the initial and final setting times remarkably and then a slight decrease for W/C ratio changing from 0.40 to 0.45. (Figure 3a,b).

The paste samples with W/C ratio of 0.30 and 0.35 displayed a constant decrease in the initial and final setting times with increasing HPMC dosage from control up to 1.5% (Figure 3c,d, Table 4). Moreover, the paste samples with W/C ratio of 0.40 and 0.45 displayed a more complex trend for both the initial and final setting time with increasing HPMC dosage (Figure 3c,d). The setting times of the pastes with W/C ratio of 0.40 and 0.45 decreased when 0.5% HPMC was added. However, when the HPMC dosage was increased from 0.5% to 1.0%, the initial and final setting times of these pastes were extended in contrast to that with W/C ratio of 0.30 and 0.35. When the HPMC dosage changed from 1.0% to 1.5%, the corresponding paste showed a decrease in the setting time.

In the case of the paste with W/C ratio of 0.4, compared to the control sample, the initial setting time was reduced by 12 min, 3 min, and 5 min, respectively by adding 0.5%, 0.75%, and 1.5% HPMC. The final setting time decreased by 11 min, 5 min, and 9 min, respectively, for 0.5%, 0.75% and 1.5% HPMC dosage, while both the initial and final setting times were extended by 6 min for 1.0% HPMC dosage (Table 4). In the case of the paste with W/C ratio of 0.45, compared to the control sample, the initial and final setting times were reduced by nearly the same amount (16 min and 15 min, respectively) by adding 0.5% HPMC; the initial and final setting times increased by nearly the same amount for 0.75%, 1.0%, and 1.5% HPMC dosage (Table 4). From Table 4, the shortened and extended time demonstrated a similar trend between the initial and final setting times with increasing W/C ratio.

**Figure 4:**
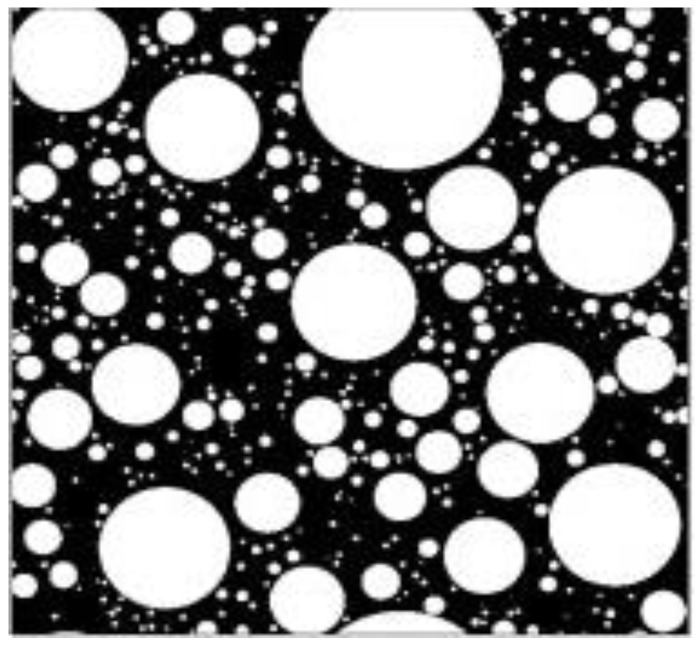
The 2D sketches of the initial paste microstructure [44].

On the basis of the numerical tool developed by Hongyan Ma et al. [43,44], 2D sketch of the early stage of the paste is shown in Figure 4. Cement particles are presented using white color, while black color represents water. Lower W/C ratio results in higher solid volume fraction and less space between particles. During the setting process of the paste, due to hydration, the interparticle space is filled, and solid phases are linked together to gradually form a dense bulk. Consequently, a paste with higher W/C ratio needs more hydration products to fill the space and form the interconnection between solid phases [45,46]. As a result, the paste with higher W/C ratio presented a longer setting time as shown in Figure 3a,b.

HPMC demonstrates can be used as a thickening admixture to increase viscoelasticity. In our tests, HPMC could shorten or extend the setting time of the freshly mixed SAC pastes with different W/C ratios and HPMC dosages measured using Vicat needle. It has been inferred that higher hydration heat release rate and higher cumulative heat evolution lead to a shorter setting time [47]. However, when the HPMC dosage was increased, the trend of hydration heat (shown in Figure 2) is not consistent with that of setting time (shown in Figure 3c,d). This phenomenon was attributed to two reasons. On the one hand, the cement particles that are covered by HPMC molecules could lead to delayed hydration and setting (as shown in Figure 2). On the other hand, HPMC exhibits gelation behavior and interlinks to construct a gel containing water with a 3D network [27]. Thus, when the thickening effect due to gelation outstrips the effect of delay in setting resulting from HPMC adsorption of water [30], it increases the resistance of the paste to the penetration of Vicat needle; therefore, the paste samples display a shorter setting time. On the contrary, when the delaying effect in the hydration outstrips the gelation effect, the pastes show an extended setting time.

**Figure 5:**
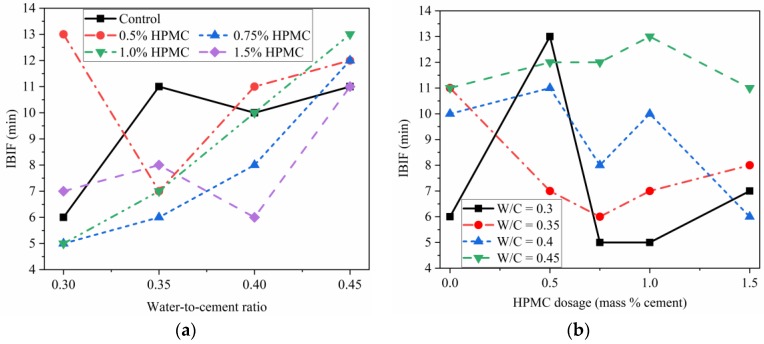
Interval between initial and final setting time (IBIF) of SAC paste with various water-to-cement ratio (**a**) and various HPMC dosages (**b**).

Figure 5 shows the intervals between initial and final setting times (IBIF) of SAC paste. The IBIF of the SAC ranged from 5 min to 13 min. The values of SAC are lower than for Portland cement [48,49]. Once the SAC paste starts losing plasticity (initial setting), it will become hardened in several minutes to hold layers on top of it. The effect of W/C ratio on the IBIF of the paste with and without HPMC is shown in Figure 5a. These results are invaluable to control the printing process, especially in the case of batch mixing, which will bring resting, including the total printing time and the time gap between the layers. The initial setting time indicates the changeover from the “flowable state” to the thixotropic state, while the final setting time reflects that the paste converts from a thixotropic state to a hardened state. The mixed material should be printed before its initial setting time. Otherwise, although the mixed material may be printable, it could result in a worse shaping, lower hardening strength, lower interlayer bond strength, and more difficult extrusion. The time gap between the deposited layers also needs to be adjusted, based on the initial setting time, final setting time and the time interval between the two. The time gap significantly influences the interlayer bond strength [37] because the deposited materials become dry with time. The cement-based bulk material produces strength due to the interconnection of the hydration products formed from cement being hydrated with water. If the surface of the printed layer becomes dry or hardened due to hydration, the interlayer bond strength will be lower.

### 3.4. Influence of W/C Ratio and HPMC Content on the Fluidity of the SAC Paste

**Figure 6:**
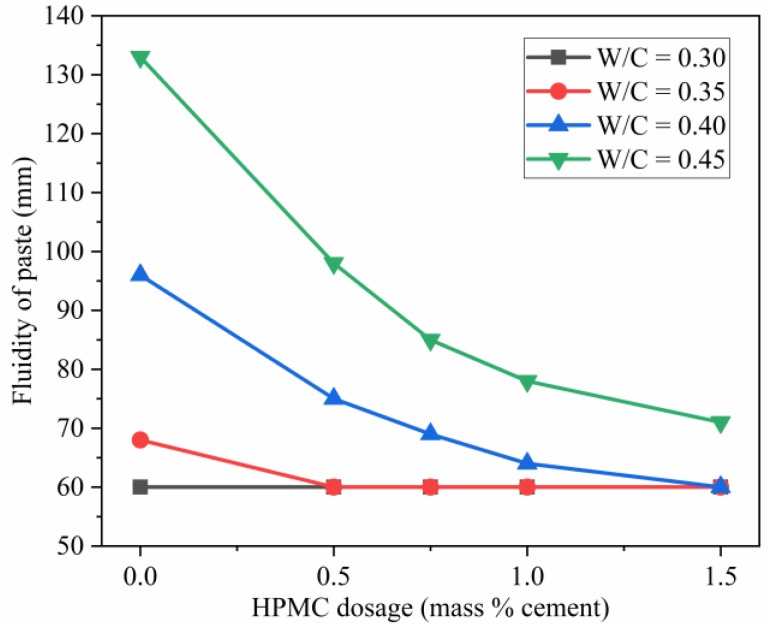
Influence of HPMC dosage and W/C ratio on fluidity of fresh SAC paste.

**Figure 7:**
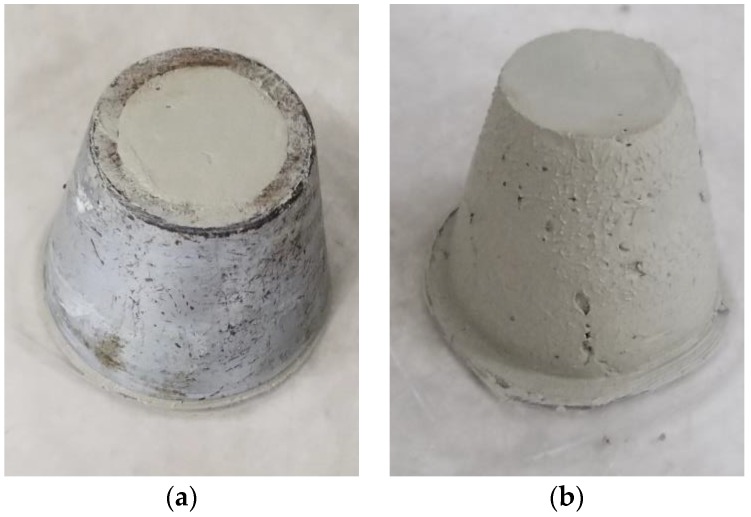
Representative pictures during measuring fluidity of paste: (**a**) a mold filled with paste; (**b**) control paste with W/C of 0.3 after removing a mold.

Figure 6 presents the influence of W/C ratio and HPMC dosage on the fluidity of the pastes. As shown in Figure 6, both W/C ratio and HPMC dosage have a significant impact on the fluidity of the SAC pastes. The lower mouth diameter of the truncated conical mold used for the fluidity test was 60 mm (Figure 7a). Thus, the test results of 60 mm fluidity showed that the pastes did not flow away after removing the mold (Figure 7b). Lower W/C ratio could result in lower paste fluidity, irrespective of the presence or absence of HPMC, because lower W/C ratio indicates smaller space and shorter distance between particles in fresh paste (Figure 4), forming greater interparticle friction. The friction is the key factor that affects fluidity. As a result, the lower the W/C ratio, the lower the fluidity. When the W/C ratio of the control paste is 0.30, the friction between the particles is high enough to withstand the flow of paste.

The results also showed that higher HPMC dosage could result in lower fluidity because 3D HPMC gel network could hold the cement particles to prevent the flow of paste. In addition, HPMC also could make the paste sticker, thus reducing fluidity. This observation is based on two reasons: the gelatinized HPMC fills the space between cement particles, and HPMC absorption of water could reduce the “virtual W/C ratio.”

Both reducing W/C ratio and increasing HPMC dosage could help cement paste maintain its molded shape. The higher friction between grains results in higher yield stress and larger grain force with decreasing W/C ratio, making extrusion harder that would need more power to print materials. Thus, incorporating HPMC and increasing HPMC dosage is a better strategy to improve shape retainability of the paste with higher W/C ratio rather than reducing W/C. It also should be noted that, even at a high W/C ratio and when removing a mold after preparation, the HPMC-SAC paste could maintain its shape.

### 3.5. Influence of S/C Ratio, W/C Ratio and HPMC Content on the Fluidity of the SAC Mortar

The initial height, final height, and fluidity were measured according to GB/T-2419–2005 using a flow table. The initial height represents the measured height of the mortar after removing the mold before vibrating. The final height represents the measured height of the mortar after being vibrated. The initial height was used to indicate the shape retainability of the mortar (standing behavior in static condition), and the final height and fluidity were used to examine the fluid behavior during transport and extrusion-based printing. Thus, the initial height of the mortar applicable to 3D printing should be as high as possible, while the corresponding final height of the mortar should be minimized, possessing maximum desired fluidity. The higher the initial height, the higher the shape retainability. The lower final height and higher fluidity indicate that the mortar has lower yield stress, and lower extrusion force is needed to print the mortar. This approach is an easy and straightforward method to assess the standing behavior of printed materials in the static condition and their fluid behavior during transportation and printing process.

**Figure 8:**
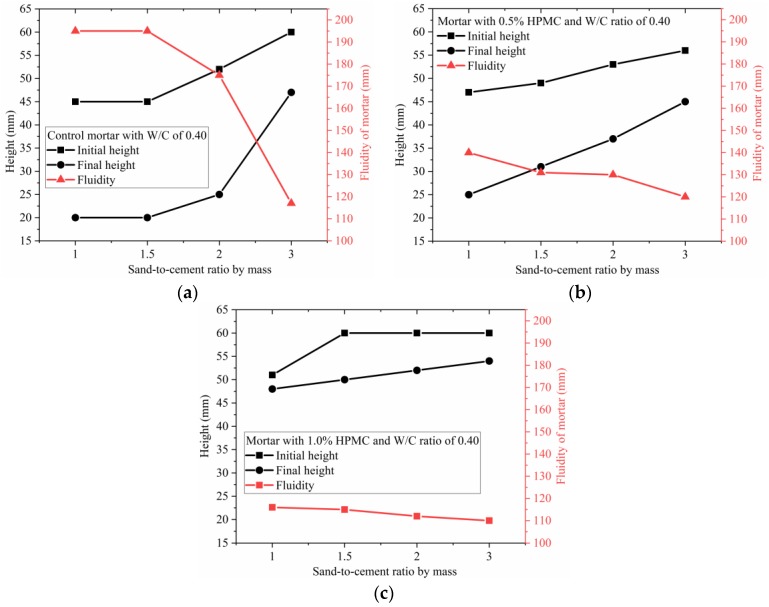
Initial height, final height and fluidity of SAC mortar Vs. S/C ratio with maintaining W/C of 0.40: (**a**) control mortar; (**b**) mortar with 0.5% HPMC; (**c**) mortar with 1.0% HPMC.

The initial height, final height, and fluidity of the mortar with W/C ratio of 0.4 and various HPMC dosages were plotted against S/C ratio, as shown in Figure 8. The increasing S/C ratio leads to an increase in initial and final heights and a decrease in fluidity. The shape retainability could be improved by increasing S/C ratio. It is likely that increasing initial and final heights, and decreasing fluidity with increasing S/C ratio are mainly attributed to greater friction between the grains, including cement particles and sand at a higher S/C ratio. When the W/C is kept constant, higher S/C ratio correlates to a larger initial solid volume fraction, and shorter intergrain distance (less intergrain space). As a result, the higher the S/C ratio, the higher the interparticle friction, both in for the static condition and during vibrating.

**Figure 9:**
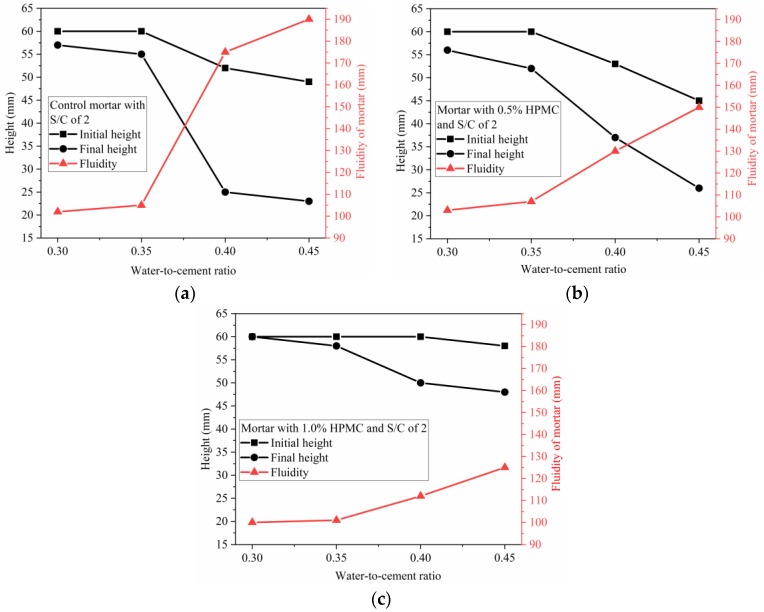
Initial height, final height and fluidity of SAC mortar Vs. W/C ratio with maintaining S/C ratio of 2: (**a**) control mortar; (**b**) mortar blended with 0.5% HPMC; (**c**) mortar blended with 1.0% HPMC.

In the case of the mortar with S/C ratio of 2, the initial height, final height, and fluidity of the mortar with various HPMC dosages were plotted against W/C ratio, as shown in Figure 9. Higher W/C ratio resulted in reducing or maintaining initial height while reducing the final height and increasing fluidity. As shown in Figure 9, the initial height of the mortar is kept constant at 60 mm with W/C ratio increased from 0.30 to 0.35 for the control mortar and 0.5% HPMC mortar, and to 0.40 for the mortar with 1.0% HPMC; however, the corresponding final height shows a continuous decline and the related fluidity continuously increases. When the W/C ratio increased, the interspace between grains (including cement particles and sand) increased due to higher initial solid volume fraction. This means lower friction between grains when the mortar tends to spread away due to gravity in the static condition or during vibration. For the control mortars with W/C ratios of 0.30 and 0.35, when they were at rest after removing the mold, the intergrain friction tended to flow away due to gravity, being successful in maintaining its shape with initial height of 60 mm; when they were vibrated, the balance between friction and gravity was disturbed, thus the mortar flowed away. Although all the mortar with W/C ratio of 0.30 had the same initial height as all the mortar with W/C ratio of 0.35, the latter showed a higher fluidity than the former due to lower intergrain friction. In comparison to the W/C ratio range between 0.30 and 0.35, the range from 0.35 to 0.40 showed a more significant influence on initial and final height, and fluidity.

**Figure 10:**
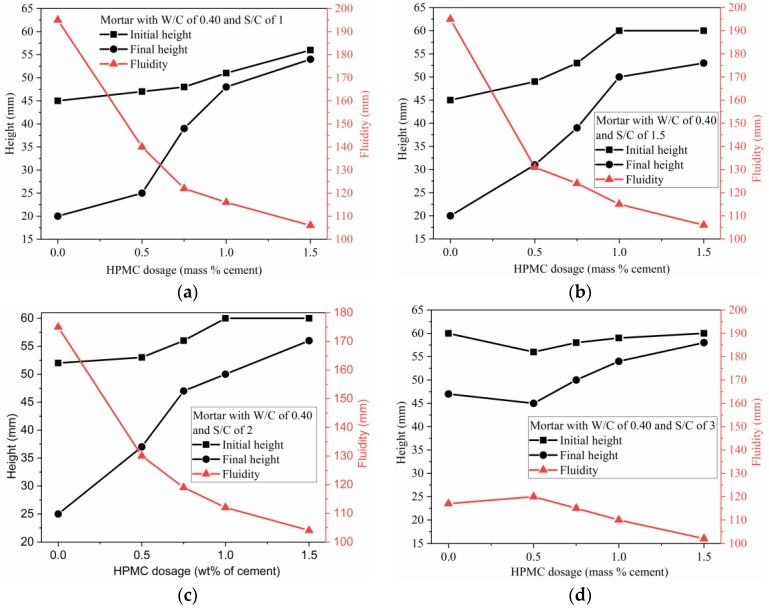
Initial height, final height and fluidity of SAC mortar Vs. HPMC dosage with maintaining W/C of 0.40: (**a**) mortar with S/C ratio of 1; (**b**) mortar with S/C ratio of 1.5; (**c**) mortar with S/C ratio of 2; (**d**) mortar with S/C ratio of 3.

The initial height, final height, and fluidity of the mortars with W/C ratio of 0.40 and various S/C ratios were plotted against HPMC dosage, as displayed in Figure 10. For the mortars prepared using W/C ratio of 0.40 and S/C ratios of 1, 1.5, and 2, as shown in Figure 10a–c, respectively, the higher HPMC dosage led to the higher initial and final heights and lower fluidity. However, for the mortar with W/C ratio of 0.40 and S/C ratio of 3, a different phenomenon occurred. The initial and final heights were first reduced by increasing HPMC dosage from 0% to 0.5%, which was followed by a continuous increase in the initial and final heights when HPMC dosage was increased from 0.5% to 1.5%. Correspondingly, there was a slight increase in fluidity that was followed by a continuous decrease. For the mortars with S/C ratio of 1, 1.5, and 2, higher HPMC dosage likely produced stronger hydrogel 3D network which provided higher resistance to spreading away. However, for the mortar with S/C ratio of 3, when HPMC dosage was between 0.5% and 1.0%, the incorporated HPMC mainly played a role as a lubricant by reducing the intergrain friction rather than blocking the fluid by the formed network. When the HPMC dosage was increased to 1.5%, the mortar maintained its shape. The result could be attributed to two reasons: the incorporated HPMC could form a stronger network, and the lubrication was not strong enough to outstrip the intergrain friction. Except for the mortar with S/C ratio of 3, as shown in Figure 10d, the other three groups showed an increasing rate in the initial height with increasing HPMC dosage from 0 to 1.0% (to 1.5% for S/C ratio of 1). This result indicates that the locking effect caused by the 3D gel network becomes stronger with increasing HPMC dosage from 0 to 1.0%.

These results are useful to modify the SAC mortar by adjusting W/C ratio, S/C ratio, and HPMC dosage, and to select the suitable mixing proportion that has high initial height, low final height and high fluidity for 3D printing. According to Figure 8, Figure 9 and Figure 10, and the analysis above, the optimal mixing proportion was found to be 1.0% HPMC, W/C ratio of 0.40 and S/C ratio of 2.

### 3.6. Influence of HPMC Content on the Early Compressive Strength of the SAC Mortar

**Figure 11:**
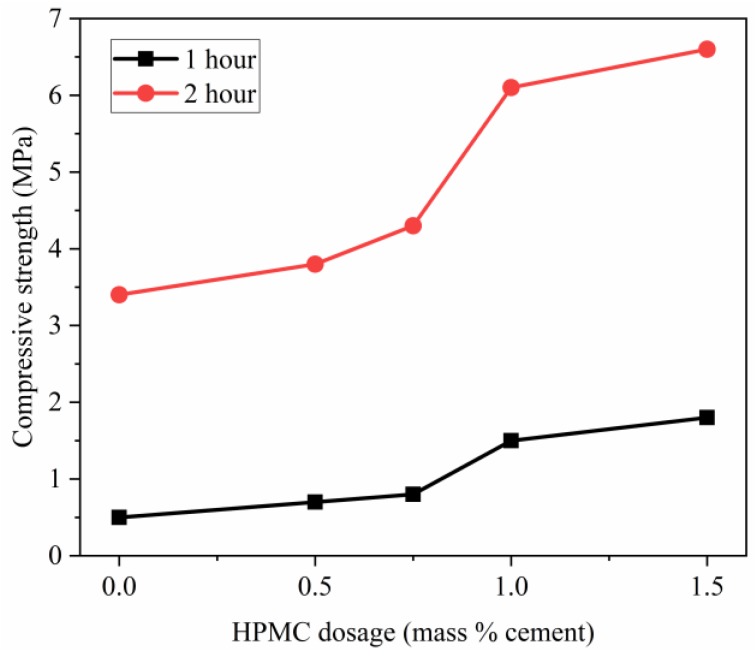
Compressive strength of mortar with W/C of 0.40, HPMC dosage of 1%, and S/C ratio of 2.

The influence of the HPMC on the early strength of the extruded mortar is shown in Figure 11. The addition of HPMC could remarkably raise the compressive strength of the extruded mortar with W/C ratio of 0.40 and S/C ratio of 2. The compressive strength of SAC mortar by extrusion molding increased from 0.5 MPa to 1.8 MPa at 1 h and from 3.4 MPa to 6.6 MPa at 2 h with increasing HPMC content from 0 to 1.5%. The addition of HPMC could make the mortar denser and more homogeneous, thereby resulting in a higher compressive strength.

### 3.7. Microstructure of Hardened SAC Paste and Mortar with and without HPMC

**Figure 12:**
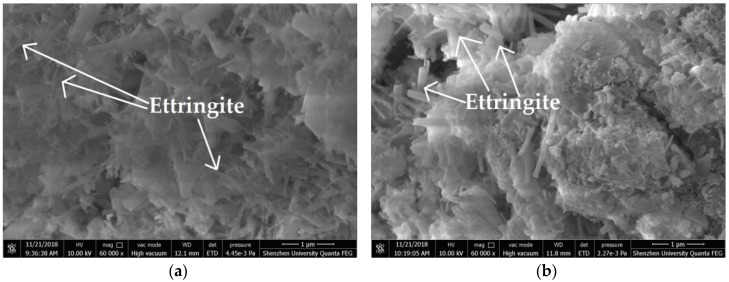
SEM images of hardened paste: (**a**) control; (**b**) with 1.0% HPMC.

**Figure 13:**
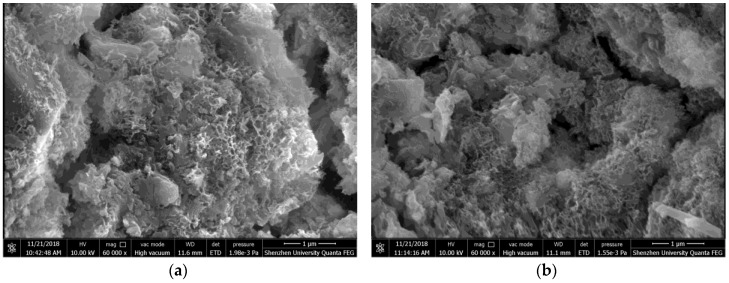
SEM images of hardened mortar: (**a**) control; (**b**) with 1.0% HPMC.

The morphology of fresh section surface of hardened SAC paste cured for 2 h was presented in Figure 12. Both the control paste and paste with 1.0% HPMC were prepared using W/C ratio of 0.40. The ettringite shown in Figure 12b had a larger size than that shown in Figure 12a, indicating that the HPMC can accelerate the growth of ettringite crystals which were produced from SAC hydration. Figure 13 shows the SEM images of section surfaces of hardened mortar with and without HPMC cured for 2 h. Both the control mortar and mortar with 1.0% HPMC were prepared using W/C ratio of 0.40 and S/C ratio of 2. There was no significant difference in microstructure between control mortar and HPMC mortar, indicating that the addition of HPMC would not affect the microstructure of SAC mortar at hydration period of 2 h.

## 4. Conclusions

HPMC was incorporated into SAC to prepare cement-based materials that can be applied for 3D printing. The following conclusions can be drawn:

1. The addition of HPMC shows a delay effect on the hydration of SAC. The delay effect was enhanced due to the increasing HPMC dosage from 0.5% to 1.0% by mass of cement. While, when the HPMC dosage increased to 1.5%, the delay effect was weakened.
2. The influence of HPMC on the setting time measured using Vicat needle depends on the W/C ratio and HPMC dosage. The HPMC composite pastes with W/C ratios of 0.30 and 0.35 have a faster setting time. While the HPMC composite pastes with W/C ratios of 0.40 and 0.45 show a decrease in the setting time with increasing HPMC dosage from 0 to 0.5%, and then an increase with increasing HPMC dosage from 0.5% to 1.0%, followed by another decrease from 1.0% to 1.5%. The reason for that is the counterbalance between the impact of hydration delay resulted from adsorption of water and gelation effect of HPMC.
3. The addition of HPMC significantly increases the viscosity of SAC paste and mortar, presenting them with both good shape retainability in the static state and fluid behavior in the kinetic state. The effect of improving shape retainability and compressive strength is mainly attributed to the gelation of HPMC, constructing a 3D network, and this network helps SAC paste and mortar show an appealing rheological behavior that could decrease or prevent flowing.
4. Furthermore, the addition of HPMC remarkably improves the early compressive strength of the mortar tested at 1 h and 2 h of hydration time.
5. The addition of HPMC accelerated the growth of ettringite crystals and had no significant influence on the microstructure of SAC mortar at hydration time of 2 h.

## Figures and Tables

**Table 1:** Chemical compositions of SAC (wt.%).

CaO	SiO_2_	Al_2_O_3_	SO_3_	MgO	Fe_2_O_3_	TiO_2_	K_2_O	Na_2_O	SrO	MnO	Others
42.3	18.4	16.1	14.5	3.63	1.27	0.64	0.473	0.25	0.117	0.086	2.234

**Table 2:** The mixing proportions (by weight) of SAC paste.

Sample No.	Cement	HPMC (wt.% Cement)	W/C
Control-0.30	1	–	0.30
Control-0.35	1	–	0.35
Control-0.40	1	–	0.40
Control-0.45	1	–	0.45
SH5-0.30	1	0.5	0.30
SH5-0.30	1	0.5	0.35
SH5-0.30	1	0.5	0.40
SH5-0.30	1	0.5	0.45
SH75-0.30	1	0.75	0.30
SH75-0.30	1	0.75	0.35
SH75-0.30	1	0.75	0.40
SH75-0.30	1	0.75	0.45
SH10-0.30	1	1.0	0.30
SH10-0.30	1	1.0	0.35
SH10-0.30	1	1.0	0.40
SH10-0.30	1	1.0	0.45
SH15-0.30	1	1.5	0.30
SH15-0.30	1	1.5	0.35
SH15-0.30	1	1.5	0.40
SH15-0.30	1	1.5	0.45

**Table 3:** The mixing proportions (by weight) of SAC mortar.

Sample No.	Cement	HPMC (wt.% Cement)	S/C	W/C
Control-1-4	1	–	1	0.40
Control-15-4	1	–	1.5	0.40
Control-2-3	1	–	2	0.30
Control-2-35	1	–	2	0.35
Control-2-4	1	–	2	0.40
Control-2-45	1	–	2	0.45
Control-3-4	1	–	3	0.40
SH5-1-4	1	0.5	1	0.40
SH5-15-4	1	0.5	1.5	0.40
SH5-2-3	1	0.5	2	0.30
SH5-2-35	1	0.5	2	0.35
SH5-2-4	1	0.5	2	0.40
SH5-2-45	1	0.5	2	0.45
SH5-3-4	1	0.5	3	0.40
SH75-1-4	1	0.75	1	0.40
SH10-1-4	1	1.0	1	0.40
SH10-15-4	1	1.0	1.5	0.40
SH10-2-3	1	1.0	2	0.30
SH10-2-35	1	1.0	2	0.35
SH10-2-4	1	1.0	2	0.40
SH10-2-45	1	1.0	2	0.45
SH10-3-4	1	1.0	3	0.40
SH15-1-4	1	1.5	1	0.40

**Table 4:** Setting time measured using Vicat needle.

W/C Ratio		Setting Time (min)	Shortened/Extended Time (min)			
		Control	0.5% HPMC	0.75% HPMC	1.0% HPMC	1.5% HPMC
0.3	Initial	32	−8	−4	−7	−14
	Final	38	−1	−8	−8	−13
0.35	Initial	43	−10	−13	−16	−21
	Final	54	−14	−18	−20	−24
0.4	Initial	50	−12	−3	+6	−5
	Final	60	−11	−5	+6	−9
0.45	Initial	56	−16	+4	+9	+3
	Final	67	−15	+5	+11	+3

Note:‘−’ means ‘shortened’,‘+’ means ‘extended’.
